# Commentary on genetic mechanisms of antimicrobial resistance in bacteria from U.S. food animals: ESBLs are here

**DOI:** 10.3389/fmicb.2013.00214

**Published:** 2013-07-30

**Authors:** Steven Roach, David Wallinga

**Affiliations:** ^1^Food Animal Concerns TrustChicago, IL, USA; ^2^Keep Antibiotics Working, St. PaulMN, USA

While Frye and Jackson do a good job describing the common mechanisms of resistance found in food animals in the U.S., they err in stating that in the U.S. extended spectrum β-lactamases (ESBLs) “thus far have only been found in human and not food animal isolates.”

In fact, Wittum et al., in 2010 first reported the collection of *Escherichia coli* expressing CTX-M-type ESBLs in fecal isolates from healthy cattle at a livestock market and from a diagnostic isolate submitted to the Ohio Animal Disease Diagnostic Laboratory. Since then, CTX-M-type ESBLs have been found in *E. coli* collected from healthy dairy calves in the western U.S. (Davis et al., 2011) and from 5 of 20 dairy farms in Ohio (Mollenkopf et al., 2012); in clinical *Salmonella enterica* isolates from swine in Minnesota and from turkeys in 4 states (Wittum et al., 2012); in *E. coli* from swine finishing barns in Michigan and Ohio and in *Klebsiella pneumoniae* from swine in Illinois (Mollenkopf et al., 2013).

We are not aware of studies finding CTX-M-type ESBLs in isolates from broiler chickens, but a retail chicken meat *E. coli* isolate expressing CTX-M-type ESBLs from Pennsylvania has been reported (Doi et al., 2010). Given the situation in other livestock species and in other countries, we expect the lack of detection of CTX-M-type ESBLs in U.S. chicken isolates is more the result of lack of appropriate studies than a real absence of these on chicken farms. In addition to CTX-M-type ESBLs, the study of dairy calves in the western U.S. also found *E. coli* expressing OXA-type ESBLs (Davis et al., 2011).
